# Predictors of outcome in patients with parvovirus B19 positive endomyocardial biopsy

**DOI:** 10.1186/1532-429X-17-S1-O80

**Published:** 2015-02-03

**Authors:** Simon Greulich, Ingrid Kindermann, Julia Schumm, Andrea Perne, Stefan Birkmeier, Stefan Grün, Peter Ong, Tim Schäufele, Steffen Schneider, Michael Böhm, Udo Sechtem, Heiko Mahrholdt

**Affiliations:** 1 Cardiology, Robert Bosch Medical Center, Stuttgart, Germany; 2 Klinik für Innere Medizin 3, Universitätsklinikum Saarland, Homburg, Germany; 3 Cardiology, Horst-Schmidt-Kliniken, Wiesbaden, Germany; 4 Medicine 2, University Medical Center of the Johannes Gutenberg University Mainz, Mainz, Germany; 5 Institut für Herzinfarktforschung Ruhr, Essen, Germany

## Background

The primary objective of this study was to establish the prognostic value of the myocardial load of PVB19 genomes in patients presenting for endomyocardial biopsy work-up of myocarditis and/or dilated cardiomyopathy in comparison to clinical, and cardiovascular MR parameters.

## Methods

108 consecutive patients who underwent EMB because of suspected myocarditis and/or dilated cardiomyopathy, and had evidence of myocardial PVB19 by PCR were enrolled. The mean follow-up was 1319 days. Primary endpoint was all-cause mortality, secondary endpoint was a composite of cardiac mortality and hospitalization for heart-failure.

## Results

Mean ejection fraction of all patients was 40%. We found n=27 patients to have a viral load ≥500 GE, n= 72 had 100-499 GE and n=9 had <100 GE. Immunohistology revealed chronic myocarditis in n=66 patients, DCM in n=17, other pathologies in n=12 (including 1 acute myocarditis), and latent PVB19 in n=13. During follow-up 11 of 108 patients died, two patients suffered SCD but were successfully shocked by their ICD, and 21 patients were hospitalized for heart failure.

Interestingly, not the viral load, but functional parameters such as LV-EF, LV-EDV (for endpoint 2), as well as the histologic diagnosis of DCM and the presence of LGE (for all endpoints) reached statistical significance. In fact, the presence of LGE yields an odds-ratio for a lethal event of 8.56 (endpoint 1), and of 5.52 for endpoint 2.

Importantly, no patient with normal LV-EF, or the absence of LGE suffered cardiac death during long-term follow-up.

## Conclusions

The viral load of PVB19 genomes in the myocardium is not related to the long-term clinical outcome. Furthermore, this study underscores the growing role of imaging parameters for risk-stratification of patients with non-ischemic myocardial disease.

## Funding

This work was funded in part by the Robert Bosch Foundation [1) clinical research grant for CMR risk stratification in HCM and 2) clinical research grant for inflammatory heart disease KKF-11-18, KKF-13-2].

